# Tissue metabolomics identified new biomarkers for the diagnosis and prognosis prediction of pancreatic cancer

**DOI:** 10.3389/fonc.2022.991051

**Published:** 2022-09-02

**Authors:** Chang Liu, Henan Qin, Huiying Liu, Tianfu Wei, Zeming Wu, Mengxue Shang, Haihua Liu, Aman Wang, Jiwei Liu, Dong Shang, Peiyuan Yin

**Affiliations:** ^1^ Key Laboratory of Integrative Medicine, the First Affiliated Hospital of Dalian Medical University, Dalian, China; ^2^ Institute of Integrative Medicine, Dalian Medical University, Dalian, China; ^3^ Department of Oncology, The First Affiliated Hospital of Dalian Medical University, Dalian, China; ^4^ iPhenome biotechnology (Yun Pu Kang) Inc., Dalian, China; ^5^ Department of General Surgery, The First Affiliated Hospital of Dalian Medical University, Dalian, China

**Keywords:** pancreatic cancer, metabolism, biomarker, prognosis, The Cancer Genome Atlas (TCGA), Genotype-Tissue Expression (GTEx)

## Abstract

Pancreatic cancer (PC) is burdened with a low 5-year survival rate and high mortality due to a severe lack of early diagnosis methods and slow progress in treatment options. To improve clinical diagnosis and enhance the treatment effects, we applied metabolomics using ultra-high-performance liquid chromatography with a high-resolution mass spectrometer (UHPLC-HRMS) to identify and validate metabolite biomarkers from paired tissue samples of PC patients. Results showed that the metabolic reprogramming of PC mainly featured enhanced amino acid metabolism and inhibited sphingolipid metabolism, which satisfied the energy and biomass requirements for tumorigenesis and progression. The altered metabolism results were confirmed by the significantly changed gene expressions in PC tissues from an online database. A metabolites biomarker panel (six metabolites) was identified for the differential diagnosis between PC tumors and normal pancreatic tissues. The panel biomarker distinguished tumors from normal pancreatic tissues in the discovery group with an area under the curve (AUC) of 1.0 (95%CI, 1.000−1.000). The biomarker panel cutoff was 0.776. In the validation group, an AUC of 0.9000 (95%CI = 0.782–1.000) using the same cutoff, successfully validated the biomarker signature. Moreover, this metabolites panel biomarker had a great capability to predict the overall survival (OS) of PC. Taken together, this metabolomics method identifies and validates metabolite biomarkers that can diagnose the onsite progression and prognosis of PC precisely and sensitively in a clinical setting. It may also help clinicians choose proper therapeutic interventions for different PC patients and improve the survival of PC patients.

## Introduction

Pancreatic cancer (PC) is one of the deadliest solid malignancies, and it has an extremely low 5-year survival rate of <5% and is expected to be the second leading cause of cancer death by 2030 (1–3). Since early symptoms of PC were not obvious and there is a lack of reliable and effective methods for early detection, over 80% of patients present with locally advanced or distant metastatic disease when the disease is clinically diagnosed (2–6). In a clinical setting, personalized therapies can be defined according to the clinical staging system and the pathological results. Besides, serum carbohydrate antigen 19-9 (CA19-9), carcinoembryonic antigen (CEA), and CA125, which are commonly used in clinical practice for PC diagnosis, have inadequate prognostic relevance (7–9). KRAS, TP53, CDKN2A, and SMAD4 are the most common somatic mutated genes in pancreatic cancer; 90% of patients have functional mutations in the KRAS oncogene, and 25%–80% of patients have functional mutations in TP53, CDKN2A, and SMAD4 oncogene (9–12). However, the mechanism of pancreatic carcinogenesis is complex, and somatic mutation is an imperfect method to rely on to comprehensively reflect metastasis and progression of PC. Therefore, the discovery of new biomarkers that can diagnose the PC more sensitively and specifically and predict prognoses in a clinical setting is urgently needed.

Metabolic reprogramming for tumorigenesis has been recognized as a hallmark of PC (13–15). After genomics, transcriptomics, and proteomics, metabolomics, the multi-omics technique that can picture the dynamic profiles of metabolism, has been recognized as a useful tool to identify novel biomarkers for an earlier diagnosis of different malignancies (15, 16). Small changes in the genome and proteome in disease states can be reflected and amplified at the metabolome level. Metabolic changes are prospective and sensitive in response to environmental perturbations, which have important potential for detecting early features prior to actual phenotypic changes. As shown in previous research, through metabolomic analysis of plasma or serum samples, researchers have revealed that nine metabolites were able to discriminate pancreatic ductal adenocarcinoma (PDAC) from chronic pancreatitis (17). Another study used the precision-targeted metabolomics method to identify and validate five new metabolite biomarkers in plasma, which can diagnose the onsite of PC progression and predict the metastasis (18). Similarly, it is worth identifying the specific features of PC metabolism that related to prognosis based on observing PC tissue metabolism, which may benefit personalized healthcare. Metabolomics has been employed to preliminarily explore biomarkers in PC diagnosis, but the relationship between metabolic reprogramming characteristics and the prognosis of PC still needs to be further explored.

Here, metabolomics was applied to unveil the metabolic signature of PC from tumor tissues with paired para-carcinoma tissues and normal pancreatic tissues. We aim to assess the performance of the tissue biomarker signature to distinguish patients with PC and to predict the prognosis of PC patients.

## Materials and methods

### Clinical samples

Matched pairs of pancreatic cancer (35 case of pancreatic cancer tissues, 34 cases of para-carcinoma tissues, and 31 cases of normal pancreatic tissues) were obtained from 35 patients undergoing curative resection at the First Affiliated Hospital of Dalian Medical University. The study was approved by the Ethics Committee of the First Affiliated Hospital of Dalian Medical University with approval number PJ-KS-KY-2021-203. All samples were freshly frozen and stored at −80°C before metabolomics analysis.

### Sample preparation for metabolomics

#### Tissue metabolites extraction

Each tissue sample was taken nearly 20 mg into the grinding pipe, and the specific weight was recorded. Then, 300 µl of methanol was added, and the grinding steel beads were put into each tube. The tissue was ground for 350 s at 120 Hz. Then, 900 µl of methyl tert-butyl ether (MTBE) was added into the tube and vortexed for 5 min, followed by the addition of 250 µl purified water; then, the mixture was shaken well for 10 min at room temperature. The mixture was kept at 4°C for another 10 min to facilitate stratification and then centrifuged at 13,000 *g* for 15 min. Finally, 700 µl of the lipid extract was transferred from the upper layer to the new centrifuge tube, and 400 µl of the polar extract was transferred from the lower layer to the other centrifuge tube.

All the remaining extracts were mixed in each sample tube evenly and then centrifuged at 10,000 *g* for 10 min. Taking the 200 µl lipid layer and 200 µl polar layer as before, they were transferred into two different 2-ml centrifuge tubes (new) and used as quality control (QC) samples. Lastly, all samples were concentrated and dried by vacuum centrifugation

#### Metabolomics data acquisition

Three different analytical methods were used for polar metabolites analysis, which was done on an Ultimate 3000 ultra-high-performance liquid chromatography and Q Exactive quadrupole-Orbitrap high-resolution mass spectrometer (Thermo Scientific, USA). Before analysis, polar extracts were accurately added with 100 µl acetonitrile–water complex solution (1:3, v/v), lipid was extracted with 80 µl acetonitrile–isopropanol solution (1:1, v/v), the mixture was vortexed for 5 min, and it was centrifuged at 13,000 *g* for 15 min (4°C). Supernates of polar extracts (90 µl) and lipid extract (70 µl) were taken for detection. The detailed methods were as described before (19).

### Metabolite panel biomarkers establishment and validation

Pathway enrichment was conducted on differential metabolites between pancreatic tumor tissues and paired normal pancreatic tissues. We also searched The Cancer Genome Atlas (TCGA) and the Genotype-Tissue Expression (GTEx) database and analyzed the expressions of key genes in the enriched Kyoto Encyclopedia of Genes and Genomes (KEGG) pathways from PC patients. Combined with the results that we achieved from the online database, we selected pathways that have both differential genes and metabolites between pancreatic tumor tissues and paired normal pancreatic tissues. Then, we chose metabolites that belong to these pathways and had log_2_ hold change >1.3 or <−1.3.

Tissue samples were randomly divided into two groups: establishment group and validation group. There were 20 normal pancreatic tissues and 20 pancreatic cancer tissues in the establishment group, while there were 11 normal pancreatic tissues and 15 PC tissues in the validation group. The least absolute shrinkage and selection operator (LASSO) algorithm analysis was used to confirm the independent predictors and build a metabolite panel for differentiating pancreatic tumor tissues and normal pancreatic tissues. The area under the curve (AUC) was used to estimate the performance of the discriminant model. The Youden index (J=sensitivity+specificity−1) was calculated in conjunction with binary logistic regression. We tested the ability of the discriminant model to predict the prognosis of PC patients by log-rank test and Cox regression. Univariate analysis was statistically significant at a p-value <0.10 and was entered into a multivariable Cox proportional-hazards model.

### Data analysis and visualization

The metabolites that were consistently detected in at least 80% of the samples were included in the statistical analysis. Multivariate statistical analyses orthogonal partial least squares discrimination (OPLS-DA) and network analysis were performed using the open-access software Metaboanalyst 4.0 (http://www.metaboanalyst.ca/MetaboAnalyst/). Transcriptomic data of PAAD samples were downloaded from the TCGA data portal (https://portal.gdc.cancer.gov/) and GTEx (https://gtexportal.org/). Gene set enrichment analysis has been performed on MSigDB Collections (https://www.gseamsigdb.org/gsea/msigdb). Unpaired t-test, binary logistic regression, log-rank test, and Cox regression analyses with OS were performed using SPSS 26.0 software (IBM, USA). Functional enrichment metabolic pathway analysis of metabolites was performed using MetaboAnalyst. The LASSO algorithm, heatmap, bar, and nomogram plots were conducted on R studio (version 3.6). Kaplan–Meier curves were conducted on GraphPad Prism 8.0. All of the p-values involved in this study were two-tailed probabilities. The difference was statistically significant for p <0.05.

## Results

### Baseline characteristics of pancreatic cancer patients

**Figure 1:**
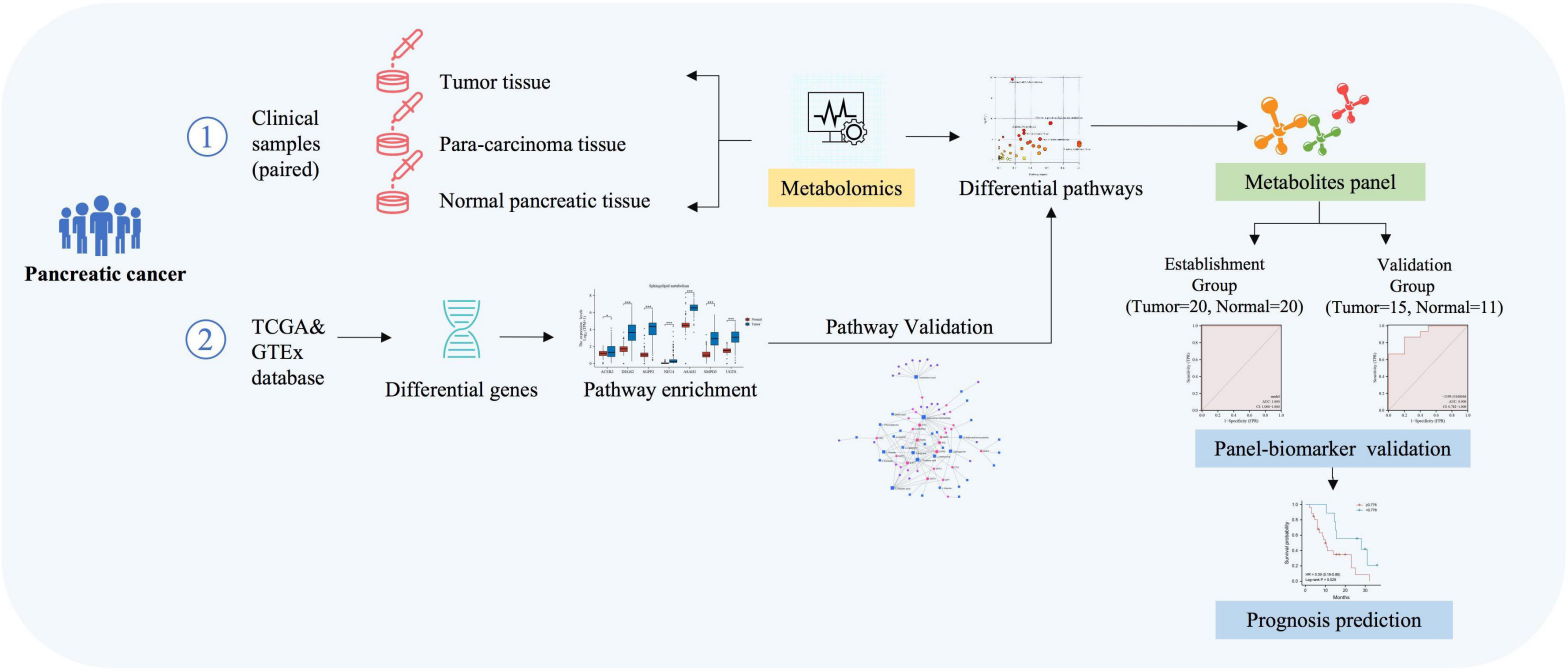
Workflow of the analysis process.

A total of 35 patients were identified from those histologically confirmed with PC and underwent resection in this study (**Figure 1**). This study included 35 PC tissues, 34 cases of para-carcinoma tissues, and 31 cases of normal pancreatic tissues. The baseline characteristics of the study cohort are set in **Table 1**.

**Table 1:** Baseline characteristics of patients with pancreatic cancer.

Characteristics	N(%)
**Gender**	
Male	18 (51.4%)
Female	17 (48.6%)
**Age**	
<60	10 (28.6%)
≥60	25 (71.4%)
**CA19-9**	
≥27 U/ml	28 (80.0%)
<27 U/ml	7 (20.0%)
**CEA**	
≥5 ng/ml	13 (37.1%)
<5 ng/ml	22 (62.9%)
**CA125**	
≥35 U/ml	10 (28.6%)
<35 U/ml	25 (71.4%)
**Tumor size**	
>3 cm	19 (54.3%)
≤3 cm	16 (45.7%)
**Differentiated degree**	
High	24 (68.6%)
Low	11 (31.4%)
**Lymph node metastasis**	
No	18 (51.4%)
Yes	17 (48.6%)
**Distant metastasis**	
Yes	7 (20.0%)
No	28 (80.0%)

### Tissue metabolomics identify significant metabolic alterations between tumors and paired normal pancreatic tissues of PC patients

**Figure 2:**
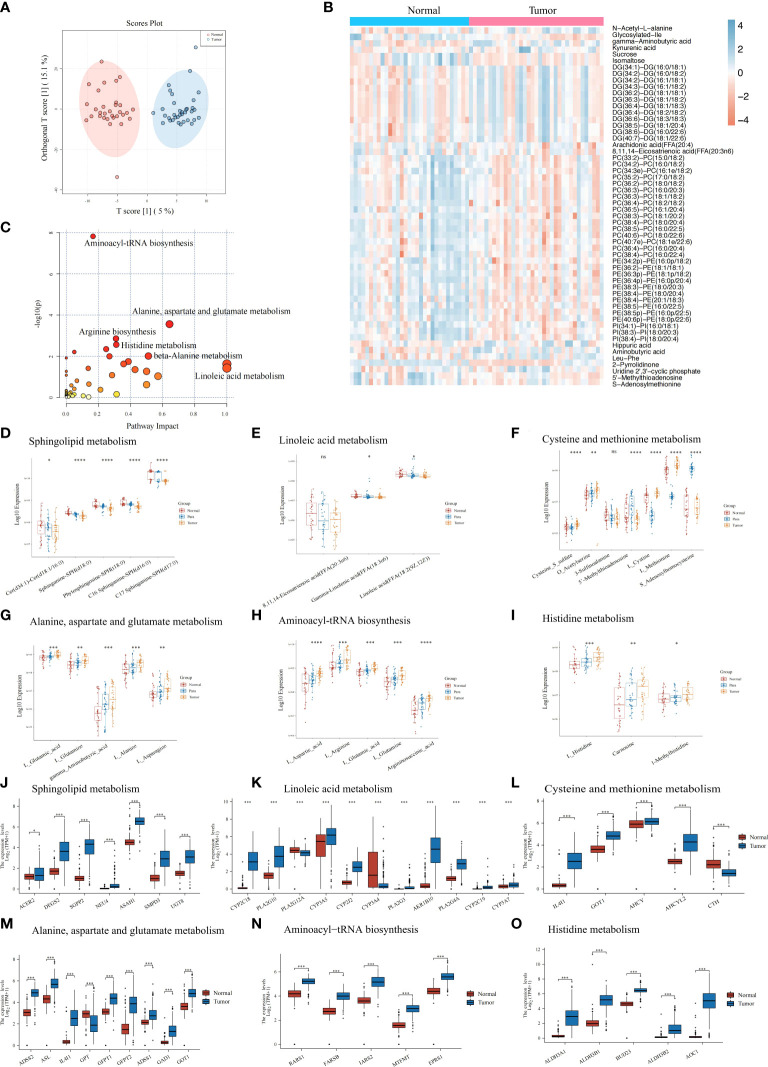
Metabolic features of the pancreatic tumor tissues, paired para-carcinoma tissues, and paired normal pancreatic tissues. **(A)** Orthogonal partial least squares discrimination analysis (OPLS-DA) score scatter plot of pancreatic tumor tissues and normal pancreatic tissues. **(B)** Heatmap of 233 with significant changes by comparing pancreatic tumor tissues and normal pancreatic tissues. Blue, increased metabolite. Orange, decreased metabolite. **(C)** KEGG pathway analysis of the 233 significantly changes metabolites mentioned above. **(D–I)** Metabolites expression in three groups (pancreatic tumor tissues, paired para-carcinoma tissues, and paired normal pancreatic tissues) involved in sphingolipid metabolism **(D)**, linoleic acid metabolism **(E)**, cysteine and methionine metabolism **(F)**, alanine, aspartate, and glutamate metabolism **(G)**, aminoacyl-tRNA biosynthesis **(H)**, and histidine metabolism **(I)**. **(J–O)** TCGA plus GTEx online results showed that genes differentially expressed in normal and tumor pancreatic tissues are also involved in sphingolipid metabolism **(J)**, linoleic acid metabolism **(K)**, cysteine and methionine metabolism **(L)**, alanine, aspartate, and glutamate metabolism **(M)**, aminoacyl-tRNA biosynthesis **(N)**, and histidine metabolism **(O)**. ****p<0.0001, ***p<0.001, **p<0.01, *p<0.05. ns, p>0.05.

Using our UHPLC-HRMS untargeted metabolomics method, the tumors tissue samples (n = 20) and paired normal pancreatic tissues (n = 20) collected from the PC patients were comparatively analyzed. Substantial metabolic changes were observed using a supervised OPLS-DA model to distinguish the tumor tissue from the normal controls sensitively. This model achieved 0.783 for Q2 (p<0.001) and 0.901 for R2Y (p<0.001) with 1,000 permutation tests (**Supplementary Figure S1A**), and the score plot depicted obvious differences between the two groups (**Figure 2A**). The QC samples in the score plot clustered tightly together, confirming the analytical reliability of the UHPLC-HRMS method used in our study (**Supplementary Figure S1B**). The heatmap showed the top metabolites (log_2_ FC >1.3 or <−1.3) that were observed to metabolic differentiate between the PC tumors tissue and the normal tissues (**Figure 2B**); details of the metabolites are listed in **Supplementary Table S1**. Then, we conducted KEGG pathway enrichment analysis (**Figure 2C**), and results showed that tumor metabolic changes occurred mainly in aminoacyl-tRNA biosynthesis, arginine biosynthesis, histidine metabolism, and other metabolic pathways. We compared the expressions of these altered metabolites from these pathways in pancreatic tumors tissue, para-carcinoma tissue, and normal tissues (**Supplementary Tables S1, S2**). These differential metabolites depicted the characteristics of pancreatic tumor from a metabolic perspective, especially dysregulated metabolites expression in sphingolipid metabolism (**Figure 2D**), linoleic acid metabolism (**Figure 2E**), and cysteine and methionine metabolism (**Figure 2F**) while upregulated metabolites expression in alanine, aspartate, and glutamate metabolism (**Figure 2G**), aminoacyl-tRNA biosynthesis (**Figure 2H**), histidine metabolism (**Figure 2I**), beta-alanine metabolism (**Supplementary Figure S1C**), arginine and proline metabolism (**Supplementary Figure S1D**), phenylalanine metabolism (**Supplementary Figure S1E**), pyrimidine metabolism (**Supplementary Figure S1F**), and glycine, serine, and threonine metabolism (**Supplementary Figure S1G**).

To understand how genes in the above pathways were modulated in PC tissues, we utilized the online subset of TCGA and GTEx database PC patients and compared the gene expressions between tumor tissues and normal pancreatic tissues. As expected, we identified a group of significantly different genes between normal and tumor tissues in the above pathways. For example, expressions of enzymes that catalyze the synthesis of sphingolipids or hydrolyze sphingolipid ceramides were significantly increased in PC tumors (**Figure 2J**), which may lead to decreased sphingosines and was consistent with the metabolic results above. In addition, differentially expressed genes in several amino acid metabolism pathways were all significantly upregulated (including linoleic acid metabolism; cysteine and methionine metabolism; alanine, aspartate, and glutamate metabolism; histidine metabolism; beta-alanine metabolism; arginine and proline metabolism; phenylalanine metabolism; and pyrimidine metabolism) (**Figures 2K–O**, **Supplementary Figures S2I–K**). This confirmed the metabolic characteristics that we demonstrated before. In linoleic acid metabolism, genes that hydrolyze low-density lipoprotein and cytochrome P450 monooxygenases significantly upregulated in PC tumors.

**Figure 3:**
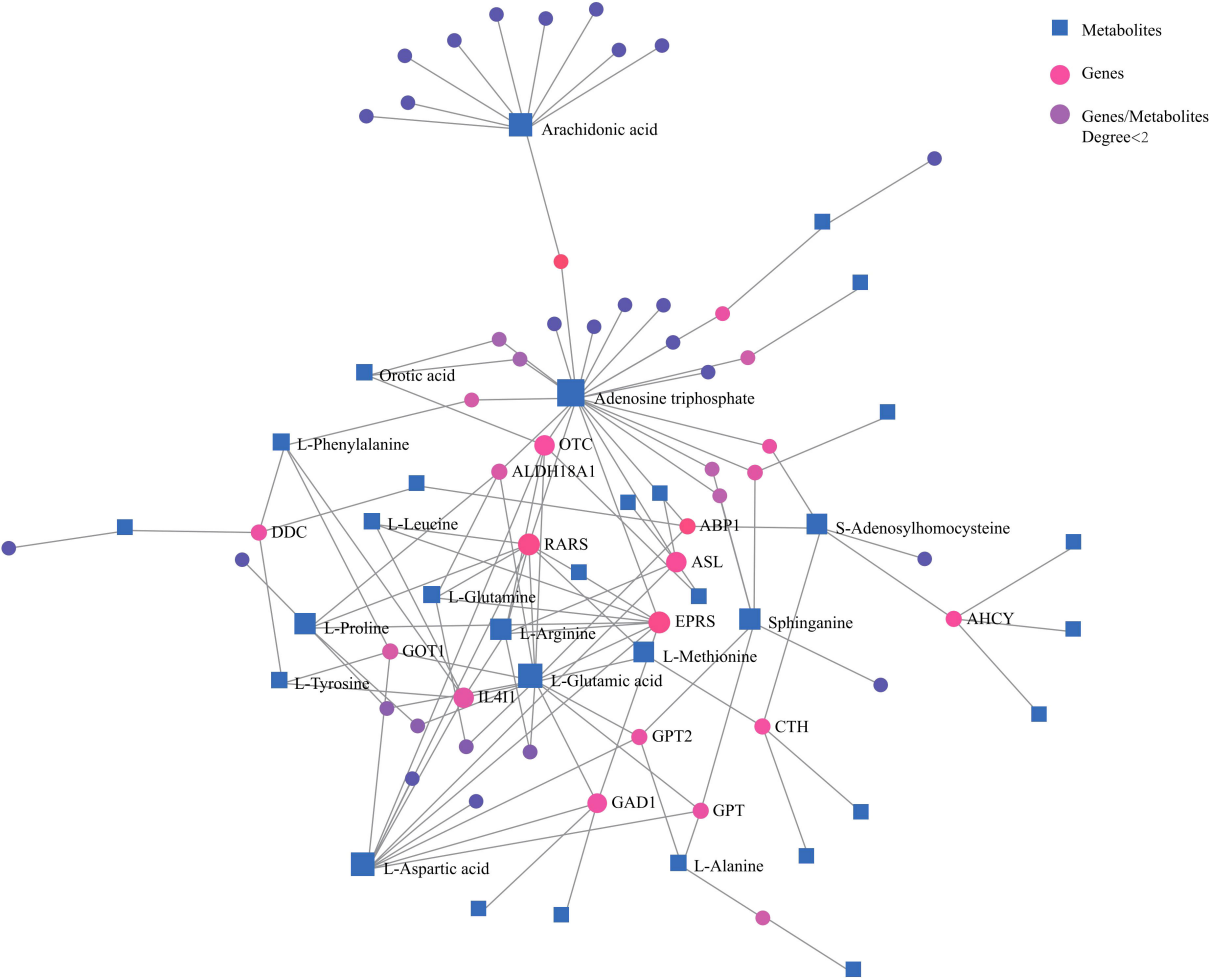
Network analysis of significantly changed metabolites and genes that are involved in key metabolic pathways. Blue square, metabolites; pink dot, genes; purple dot, genes or metabolites that did not have close relationships with others were excluded.

Network analysis of differential metabolites and genes also demonstrated correlations among important amino acids and lipids with genes (**Figure 3**). These confirmed that amino acid and lipid metabolisms played vital roles in pancreatic cancer.

**Figure 4:**
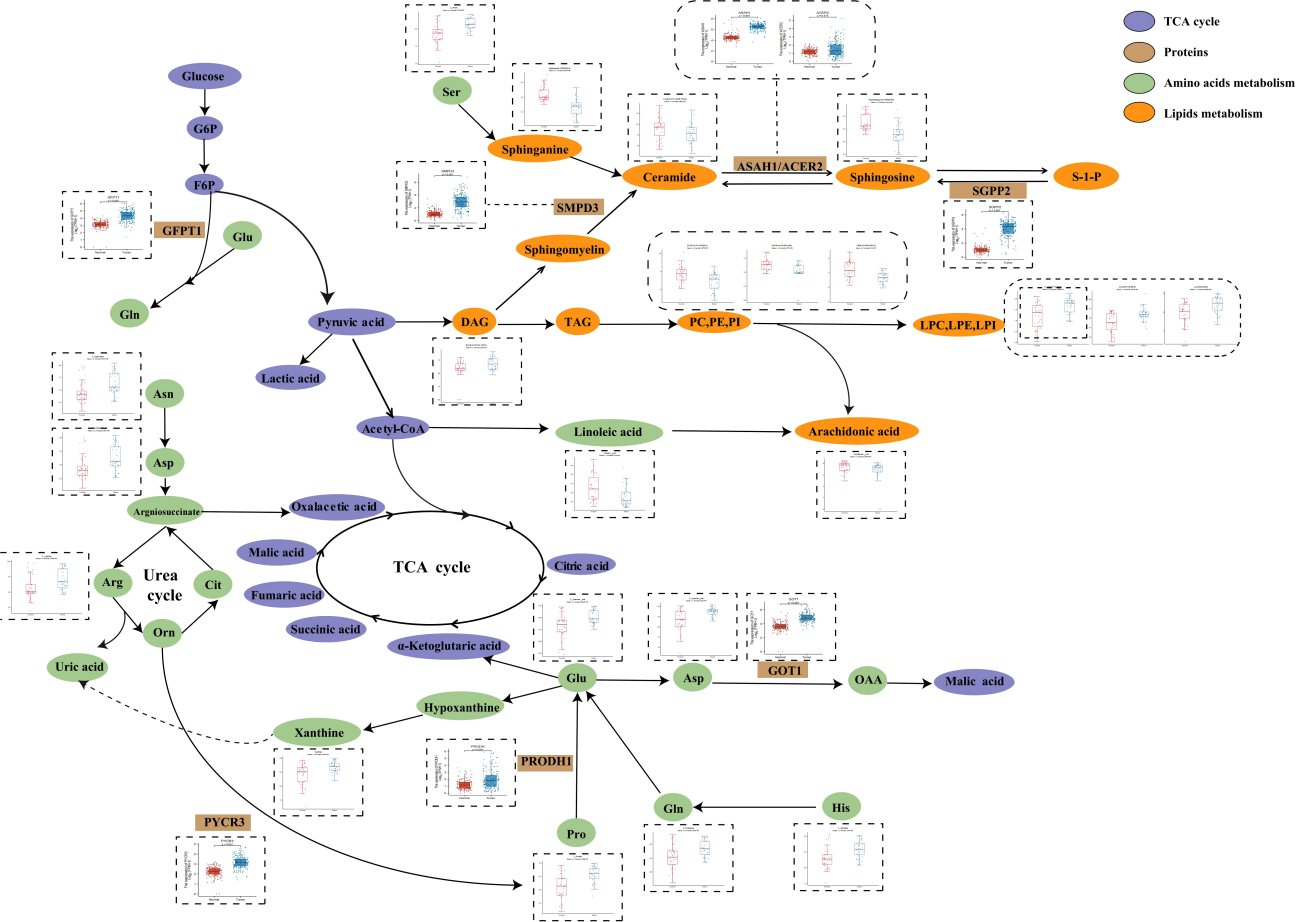
Metabolic pathways of some significantly changed metabolites and related proteins encoded by significantly changed genes in PC. The blue and red bars represent the corrected responses in the pancreatic tumor tissues and normal pancreatic tissues, respectively; purple, metabolites in TCA cycle. Brown, proteins that catalyze different metabolic changes; green, metabolites involved in amino acids metabolism; orange, metabolites involved in lipids metabolism.

In order to further investigate PC metabolic reprogramming, we provided a metabolic pathway map to illustrate alterations between tumor and normal pancreatic tissues and was constructed based on the differential metabolites and related genes (**Figure 4**). There were decreased levels of sphingolipids while there were also increased levels of key enzymes including sphingosine-1-phosphate phosphatase 2 (SGPP2), acid ceramidase (ASAH1), and alkaline ceramidase 2 (ACER2), indicating the disturbance of sphingolipid metabolism. Moreover, we observed enhanced conversion from glycerophospholipids (PC, PE, and PI) to lysoglycerophospholipids (LPC, LPE, and LPI). Serine was the source of one carbon unit, and it showed increased abundance in tumor tissues, the function of which was to accelerate fueling on synthesis of purine and pyrimidine. Hypoxanthine was oxidized to xanthine under the catalysis of xanthine oxidoreductase and subsequently converted to uric acid. The accumulation of key urea cycle metabolites (asparagine, arginine, and aspartate) and xanthine suggested the abnormal activation of urea cycle. Combined with the increased levels of pyrimidines, these in turn support the synthesis of RNA and DNA of pancreatic cancer cells and the progression of cancer.

### Metabolite panel biomarkers to evaluate pancreatic cancer

CA19-9, CA125, and CEA are clinically used as biomarkers to diagnose PC, but these biomarkers sometimes lack sensitivity and specificity (**Supplementary Figure S2A**).

**Figure 5:**
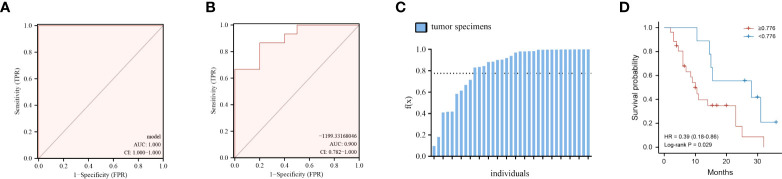
Performance of the six-metabolite panel biomarkers in the discrimination and predicting prognosis of PC. **(A, B)** ROC curve of metabolites panel model for prediction in discovery group **(A)** and validation group **(B)**. **(C)** Logistic regression values f(x) for all the patients under the discriminant models. **(D)** Kaplan–Meier curves of overall survival for all patients using the discriminant model. Red line, patients with f(x) ≥ 0.776; blue line, patients with f(x) < 0.776.

Finding metabolite panel biomarkers for precise diagnosis has a decisive impact on patient treatment and survival. According to the pathway enrichment results, we first randomly separated the tumor and normal tissue samples into two groups, namely, the model establishment group and validation group (**Supplementary Tables S3, S4**). Then, we selected significantly changed metabolites that belong to the metabolic pathways mentioned above (log_2_ fold change >1.3 or < −1.3). The details of these significantly different metabolites are listed in **Supplementary Table S1**. Furthermore, the six optimal metabolic features were selected by using the LASSO algorithm (**Supplementary Figure S2B**). To evaluate the discrimination effect of this panel, we used binary logistic regression and finally obtained the discriminant model consisting of kynurenic acid, gamma-aminobutyric acid, PC(36:2)-PC(18:0/18:2), hippuric acid, uridine 2′,3′-cyclic phosphate, and 5′-methylthioadenosine. The six metabolites discriminant models’ logistic regression values g(z) = (40.117*kynurenic acid + 1.865*gamma-aminobutyric acid − 0.071*PC(36:2)−PC(18:0/18:2) + 1.959*hippuric acid + 43.226*uridine 2′,3′-cyclic phosphate − 16.702*5′-methylthioadenosine)/100,000 − 99.161 were obtained. According to binary logistic regression, the ROC analysis of the establishment group model using the six metabolites panel yielded an AUC of 1.0 (95%CI: 1.000−1.000) (**Figure 5A**). Based on this, the discriminant model was established according to the logistic regression values g(z). To validate the accuracy of the discriminant model, logistic regression values g(z) were obtained for the patients in the validation group. The discriminant model set from the establishment group distinguished the specimens of PC from those of normal tissues in the validation set with an AUC of 0.9000 (95%CI = 0.782–1.000) (**Figure 5B**). These indicated that the metabolites panel biomarkers were significantly associated with overall survival of pancreatic cancer.

### The metabolites discriminant model is associate with the overall survival of pancreatic cancer

We then thought to determine if the metabolite panel correlates with clinical outcomes, focusing on overall survival (OS) for PC patients. First, logistic regression values g(z) for all the 35 patients, both in the discovery and validation set, were obtained according to the six-metabolite discriminant model (**Figure 5C**). The cutoff value was set as 0.776, which corresponded to the maximum Youden index of our discrimination model. The median overall survival (mOS) of patients who were f(x)<0.776 was 28.0 months, significantly longer than 10.0 months for patients with f(x)≥ 0.776 (HR=2.55, 95%CI 1.16–5.61 p= 0.029, **Figure 5D**).

We further performed a multivariate analysis of the discriminant model with other clinical factors. The COX regression results indicated that the discriminant model <0.776 and no distant metastasis were shown to be independent predictors of OS (HR= 0.161, 95%CI 0.034–0.756, p=0.021) (HR= 0.257, 95%CI 0.081–0.813, p=0.021) (**Table 2**).

**Table 2:** Univariate and multivariate analysis for overall survival of PC patients.

	HR (95% CI)	p-value
**Univariate analysis**		
Model value (<0.776 *vs*. ≥0.76)	0.355 (0.137–0.920)	0.033
Gender (female *vs*. male)	0.491 (0.214–1.126)	0.093
Age(≥60 *vs*. <60)	1.777 (0.723–4.364)	0.210
CA19-9 (U/ml) (≥27 *vs*.< 27)	1.826 (0.535–6.239)	0.337
Tumor size (≤3 cm *vs*. >3 cm)	0.699 (0.296–1.649)	0.413
Differentiated degree (low *vs*. ≥high)	0.774 (0.315–1.896)	0.575
Lymph node metastasis (absent *vs*. present)	0.930 (0.404–2.141)	0.865
Distant metastasis (absent *vs*. present)	0.369 (0.149–0.909)	0.030
**Multivariate analysis**		
Model value (<0.776 *vs*. ≥0.776)	0.161 (0.034–0.756)	0.021
Gender (female *vs*. male)	0.628 (0.254–1.552)	0.313
Age(≥60 *vs*. <60)	1.836 (0.617–5.465)	0.275
CA19-9 (U/ml) (≥27 *vs*.< 27)	0.949 (0.227–3.970)	0.943
Tumor size (≤3 cm *vs*. >3 cm)	1.321 (0.455–3.836)	0.608
Differentiated degree (low *vs*. ≥high)	1.003 (0.274–3.672)	0.996
Lymph node metastasis (absent *vs*. present)	0.920 (0.340–2.490)	0.869
Distant metastasis (absent *vs*. present)	0.257 (0.081–0.813)	0.021

## Discussion

In summary, we have uncovered the metabolic pathway alterations and the changing pattern among pancreatic tumors tissue, para-carcinoma tissue, and normal pancreatic tissues. We identified a metabolite panel of biomarkers [kynurenic acid, hippuric acid, gamma-aminobutyric acid, PC(36:2)-PC(18:0/18:2), uridine 2′,3′-cyclic phosphate, and 5′-methylthioadenosine) that can precisely diagnose pancreatic cancer by efficiently distinguishing between pancreatic tumors tissues and normal pancreatic tissues. Importantly, the metabolite panel was significantly associated with the overall survival of PC patients and can serve as a good prognostic marker for PC.

Earlier studies demonstrated that reprogrammed glucose, amino acid, and lipid metabolism in the tumor microenvironment and metabolic crosstalk contribute to the unlimited progression of pancreatic tumors (20–23). Enhanced lipid synthesis or uptake contributes to rapid cancer cell growth and tumor formation (21, 24–26). In this work, significantly increased levels of glycerophospholipids especially glycerophosphocholine in tumor tissues suggested the acquisitive demand for energy from cancer cell and the subsequent altered lipid metabolism to maintain viability and/or growth of cancer cells. Meanwhile, we also noticed inhibited sphingolipid metabolism in PC tumors with gradually decreasing sphingolipids levels from normal pancreatic tissue, para-carcinoma tissue, to tumor tissue. More recently, the role of sphingolipids in carcinogenesis and cancer treatment has been investigated, and they are becoming the novel subject for anti-cancer therapies (27–30), and the removal instead of synthetic sphingolipids eliminates cells to provide carbon and reduces their anti-cancer capacity.

The widely rewired amino acids metabolism was also observed in pancreatic tumor tissue and para-carcinoma tissue when compared with normal pancreatic tissue. Emerging evidence has revealed that amino acid metabolism plays an important role in PC initiation and progression (31–33). Consistent with former studies, several amino acid transporters were also found to be highly expressed in PC tumor tissues to satisfy the increased need for proliferation (**Figure 3B**), such as cytoplasmic aspartate transaminase (GOT1) (34), proline oxidase (PRODH1) (35), and glutamine fructose 6-phosphate amidotransferase-1 (GFPT1) (36–38).

Combining the metabolites and gene pathway enrichment results, we selected six metabolites as a panel to be a discriminant model. The panel contained metabolites from different chemical classes including lipid, amino acid, pyrimidine, and microbial metabolites. The metabolites panel achieved an ideal differentiating effect between the tumor and normal pancreatic tissue. This is suggestive of the reliability and representativeness of the select metabolites in the panel, which could be what underlies the tumorigenesis of PC. Normally, PC patients who undergo radical surgical resection had a comparable better OS, with 5-year OS rate reaching 20%. In our study, PC patients have f(x)<0.776 (the settled cutoff value based on our discriminant model); the mOS was 28.0 months and intriguingly comparable with that of radical surgical resection. These results demonstrated that our discriminant model had clinical predictive value for PC patients. However, this measurement based on tissue samples is not applicable for those who did not receive surgical resection or are on drug therapy. Therefore, it is still necessary to integrate widely accepted clinical parameters and biomarkers for a comprehensive prognostic risk assessment.

In this study, metabolic classification based on the metabolic profiles of PC tissues provided novel insights for the heterogeneity of PC and could be performed as a new method to explore the prognosis. Additionally, the association of the metabolites panel with OS of pancreatic cancer patients indicated its potential in guiding medication and treatment, which may have the capacity to monitor PC from a metabolic view and guide clinicians in PC prevention and treatment.

## Data availability statement

The original contributions presented in the study are included in the article/**Supplementary Material**. Further inquiries can be directed to the corresponding authors.

## Ethics statement

The studies involving human participants were reviewed and approved by the Ethics Committee of the First Affiliated Hospital of Dalian Medical University. The patients/participants provided their written informed consent to participate in this study. Written informed consent was obtained from the individual(s) for the publication of any potentially identifiable images or data included in this article.

## Author contributions

JL, SD, and PY conceived and designed the experiment. CL, HQ, MS, and HHL performed the experiments. HYL, TW, ZW, and AW analyzed the data CL. HQ drafted the manuscript. JL, SD, and PY reviewed the manuscript. CL and PY edited the final manuscript. All authors contributed to the article and approved the submitted version.

## Funding

This work was supported by the key research and development project of Liaoning Province (No. 2018225054), National Key Research and Development Program of China (No. 2018YFE0195200), the National Natural Science Foundation of China (No. 81873156), and Young Science and Technology Talent Project of the Education Department of Liaoning Province China (No. LZ2020075).

## Acknowledgments

We thank the Key Laboratory of Integrative Medicine, the First Affiliated Hospital of Dalian Medical University, for experiment support and the iPhenome Biotechnology (Yun Pu Kang) Inc., Dalian for data generation.

## Conflict of interest

ZW and PY are co-founders of iPhenome (Yun Pu Kang) Biotechnology Inc. ZW is an employee of iPhenome (Yun Pu Kang) Biotechnology Inc.

The remaining authors declare that the research was conducted in the absence of any commercial or financial relationships that could be construed as a potential conflict of interest.

## Publisher’s note

All claims expressed in this article are solely those of the authors and do not necessarily represent those of their affiliated organizations, or those of the publisher, the editors and the reviewers. Any product that may be evaluated in this article, or claim that may be made by its manufacturer, is not guaranteed or endorsed by the publisher.
