# Supplementary figures and images for: Tissue metabolomics identified new biomarkers for the diagnosis and prognosis prediction of pancreatic cancer
Source: Front Oncol. 2022 Sep 2;12:991051. doi: 10.3389/fonc.2022.991051 (PMC9479084; doi:10.3389/fonc.2022.991051)

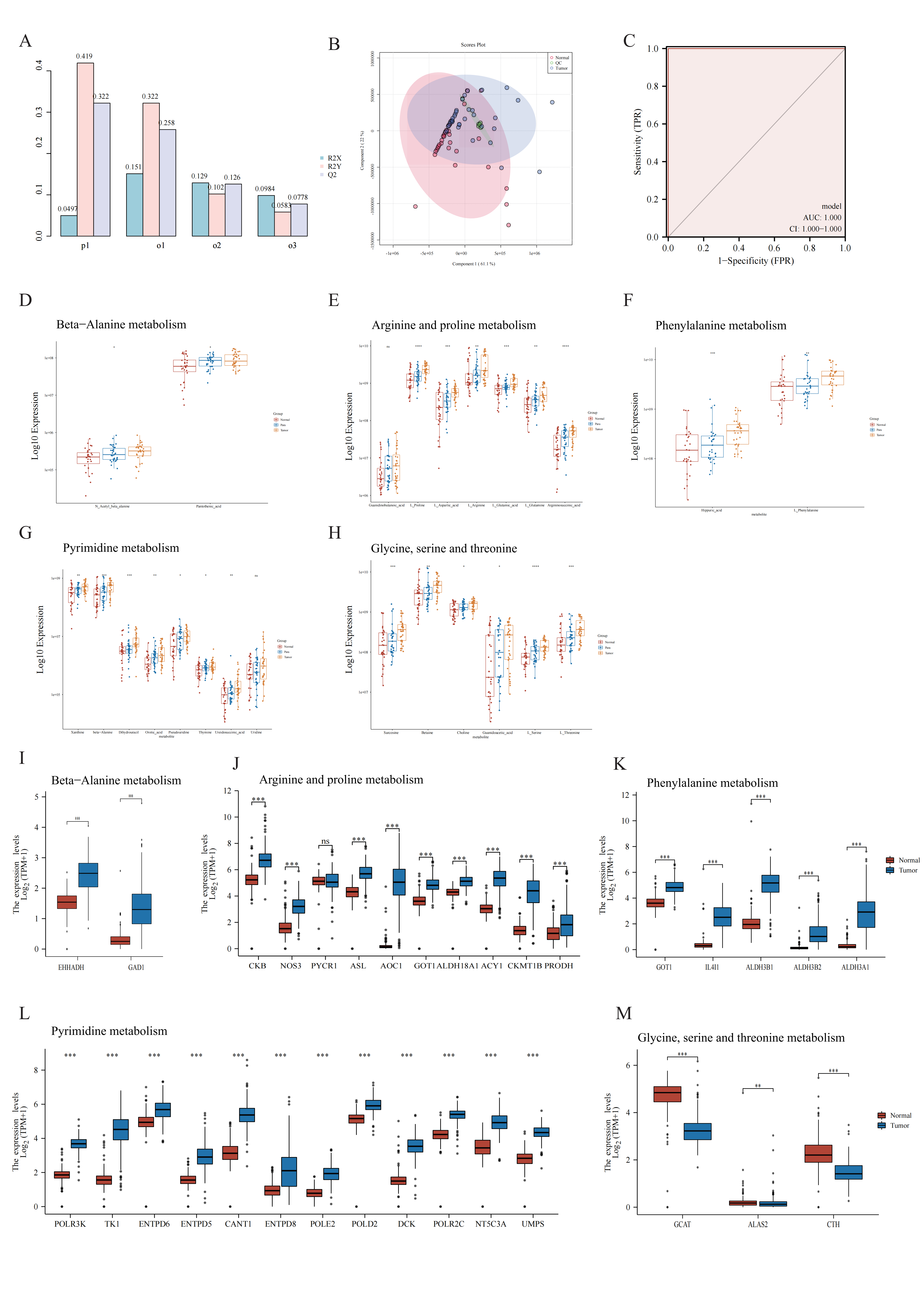

Supplement: Supplementary file 2 [file Image_1.png]

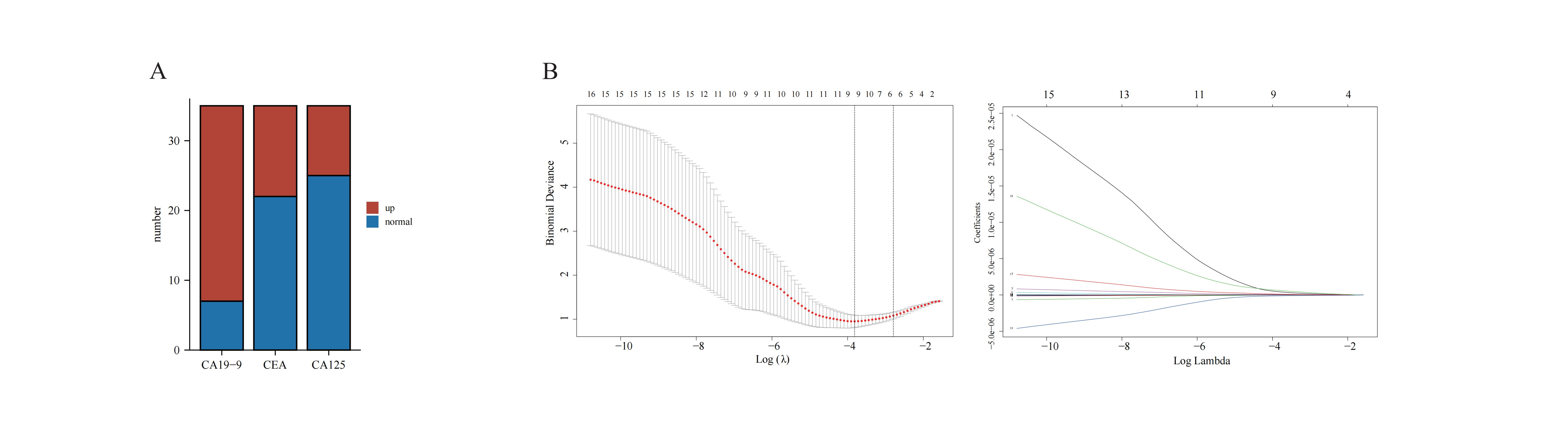

Supplement: Supplementary file 3 [file Image_2.png]
